# Cost-Effectiveness of Youth-Friendly Health Services in Health Post Settings in Jimma Zone, Ethiopia

**DOI:** 10.3390/ijerph22081179

**Published:** 2025-07-25

**Authors:** Geteneh Moges Assefa, Muluken Dessalegn Muluneh, Sintayehu Abebe, Genetu Addisu, Wendemagegn Yeshanehe

**Affiliations:** 1 Amref Health Africa, P.O. Box 20855, Addis Ababa 1000, Ethiopia; mulusef@yahoo.com (M.D.M.); sintayehu.abebe@amref.org (S.A.); genetu.addisu@amref.org (G.A.); 2 Selam Global Health Consultancy, 6701 BH Wageningen, The Netherlands; wendaab@gmail.com

**Keywords:** youth-friendly health services, Health Extension Workers, cost-effectiveness, primary healthcare, adolescent health, Ethiopia

## Abstract

Background: Adolescents in Ethiopia, particularly in rural areas, face significant barriers to accessing comprehensive sexual and reproductive health (SRH) services, resulting in poor health outcomes. The youth-friendly health services (YFHS) initiative addresses these challenges by training Health Extension Workers (HEWs) to deliver tailored, age-appropriate care at the primary care level. This study evaluates the cost-effectiveness of YFHS implementation in rural health posts in the Jimma Zone, Ethiopia. Methods: Using an ingredient-based costing approach, costs were analyzed across six health posts, three implementing YFHS and three offering routine services. Health outcomes were modeled using disability-adjusted life years (DALYs) averted, and incremental cost-effectiveness ratios (ICERs) were calculated. Results: Results showed that YFHS reached 9854 adolescents annually at a cost of USD 29,680, compared to 2012.5 adolescents and USD 7519 in control sites. The study showed the ICER of USD 25.50 per DALY averted. The intervention improved health outcomes, including a 27% increase in antenatal care uptake, a 34% rise in contraceptive use, and a 0.065% reduction in abortion-related mortality, averting 52.11 DALYs versus 26.42 in controls. Conclusions: The ICER was USD 25.50 per DALY averted, well below Ethiopia’s GDP per capita, making it highly cost-effective by WHO standards. Scaling YFHS through HEWs offers a transformative, cost-effective strategy to advance adolescent SRH equity and achieve universal health coverage in Ethiopia.

## 1. Introduction

This study is guided by human capital theory, which provides a powerful framework to understand the broader societal impact of adolescent sexual and reproductive health (SRH) interventions beyond traditional health metrics. Early pregnancy and unmet SRH needs significantly disrupt girls’ educational attainment and future economic productivity, perpetuating cycles of poverty and gender inequality. By framing youth-friendly health services as critical investments in human capital development, this analysis highlights their potential to improve not only immediate health outcomes but also long-term socioeconomic gains. This perspective underscores the importance of integrating adolescent SRH interventions within national development priorities, demonstrating their role in fostering educational completion, enhancing lifetime earnings, and contributing to sustainable economic growth [1,2,3,4].

Adolescents and youth (ages 10–24 years) represent a significant proportion of Ethiopia’s population, comprising approximately 25% of the total [5,6]. Despite their demographic prominence, young people in Ethiopia continue to experience substantial barriers in accessing health services, particularly for sexual and reproductive health (SRH) [5,7,8]. The challenges are compounded by socio-cultural taboos, inadequate provider training, poor service infrastructure, and limited awareness about available services, leading to persistently high rates of adolescent pregnancies, sexually transmitted infections (STIs), and maternal morbidity and mortality among youth [9,10,11].

A 2022 systematic review found that 78% of CHW programs were cost-effective for conditions like malaria and maternal health, demonstrating high returns by reducing facility costs and expanding rural access, though gaps remain in costing methods and sustainability analyses [12]. For adolescent health, a 2023 scoping review showed school-based SRH and HIV prevention programs in SSA had ICERs well below WHO thresholds, with integrated services improving cost efficiency; however, evidence is limited for marginalized youth [13]. Importantly, a 2021 review of CHW-led adolescent health initiatives found these programs reduced costs by 30–60% compared to clinic models while maintaining outcomes, driven by task-shifting, reduced travel, and community ownership, though economic data on managing adolescent non-communicable diseases remain scarce [14].

In recognition of these challenges, Ethiopia launched the National Adolescent and Youth Health Strategy (2021–2025), which emphasizes equity, quality, and rights-based approaches to improving adolescent and youth health outcomes. The strategy specifically calls for expanding youth-friendly health services (YFHS) at the community level, particularly through Health Extension Workers (HEWs), who are central to the country’s Health Extension Program [15]. This policy aligns with global evidence that youth-focused SRH interventions—when delivered in a culturally sensitive and accessible manner—can significantly improve service utilization and health outcomes [16].

The central question addressed by this study is whether delivering youth-friendly health services (YFHS) through Health Extension Workers (HEWs) at the primary care health post level in rural Ethiopia is cost-effective compared to standard healthcare delivery models. This question is pertinent for resource-limited settings such as Ethiopia, where maximizing health gains from limited financial investments is essential. Some international studies indicate that youth-targeted SRH interventions can be both effective and economically viable [17,18,19,20]. For instance, a study in Moldova demonstrated that integrating youth-friendly services within existing health structures significantly improved youth health outcomes and proved cost-effective [17]. Similarly, evidence from sub-Saharan Africa suggests that youth-focused health initiatives—especially those involving trained healthcare providers and dedicated infrastructure—can substantially enhance service utilization and health outcomes [21,22,23].

Most Ethiopian studies to date have focused on descriptive assessments or service utilization trends, without assessing cost-effectiveness or measuring outcomes in terms of disability-adjusted life years (DALYs) averted [24,25,26]. The absence of DALY-based evaluations represents a critical limitation in previous research, hindering cross-comparability and policy relevance. Furthermore, these studies have not sufficiently captured the differential impact of YFHS on subgroups such as rural adolescents, out-of-school youth, and married adolescent girls, thereby limiting the granularity needed for targeted interventions [25,26,27].

A critical knowledge gap exists regarding the economic viability of scaling up YFHS through Ethiopia’s extensive Health Extension Program (HEP). There is uncertainty about whether targeted adolescent SRH services at rural health posts, staffed by HEWs, could offer measurable improvements in health outcomes in a cost-effective manner. This uncertainty has implications for resource allocation decisions at national and regional levels, especially given the limited fiscal space and competing health priorities.

This study fills a key gap in the literature by conducting the first DALY-based cost-effectiveness analysis of delivering youth-friendly SRH services through HEWs at the health post level in rural Ethiopia. By integrating primary data with decision-analytic modeling, the study provides actionable, localized economic evidence to guide national adolescent health programming. The objective of this study is to determine the cost-effectiveness of providing youth-friendly health services at the health post level through trained HEWs in Ethiopia. The findings will provide essential economic evidence to inform strategic decisions for scaling adolescent health interventions within the national health system.

## 2. Materials and Methods

### 2.1. Study Design

This study employed a cost-effectiveness analysis (CEA) framework using a decision-analytic model to compare the provision of youth-friendly health services (YFHS) by Health Extension Workers (HEWs) at rural health posts with standard service delivery in Ethiopia. A decision tree model was constructed using TreeAge Pro software (2025 edition) to simulate the costs and health outcomes associated with each delivery approach [27]. The model assessed incremental cost-effectiveness ratios (ICERs), calculated as the incremental cost per disability-adjusted life year (DALY) averted, comparing the intervention against standard service delivery. The time horizon for the analysis was one year, reflecting the duration of the intervention.

#### 2.1.1. Decision Tree Model Structure

The decision tree model included branches representing service delivery pathways under both intervention and control arms. For each arm, nodes captured access to SRH services (yes/no), uptake of contraception or ANC services (yes/no), occurrence of pregnancy or STI (yes/no), and outcomes (DALYs averted due to prevented adolescent pregnancies, reduced STI incidence, and improved antenatal care (ANC) coverage). Each pathway was assigned probabilities and costs derived from field data, the literature, and national statistics. Health outcomes were converted into DALYs averted using standard Global Burden of Disease (GBD) metrics. Outcomes and costs were aggregated to derive incremental cost-effectiveness ratios (ICERs), defined as the additional cost per DALY averted by YFHS compared to standard care.

**Figure 1:**
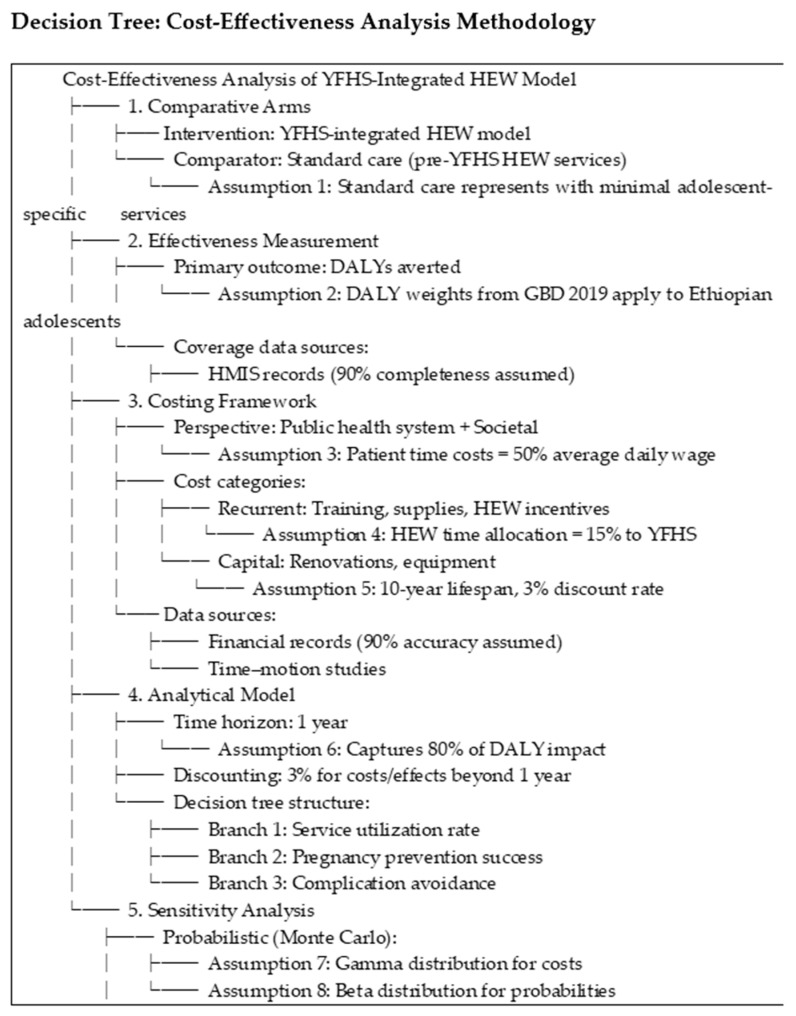
Decision tree framework for cost-effectiveness analysis of youth-friendly health services integrated into Ethiopia’s Health Extension Program.

The key assumptions used in the TreeAge decision-analytic model includes the assumed duration of contraceptive effectiveness (e.g., 3 months for injectables, 5 years for implants); probabilities of progression from untreated STIs to more severe health outcomes, including associated disability weights; and a 3% discount rate applied to future costs and outcomes, in line with standard health economic evaluation practices [12,19]. The decision tree structure (Figure 1) guided our cost-effectiveness comparison of the YFHS-integrated HEW model versus routine care. We adopted a public health system and societal perspective, incorporating key parameters of effectiveness (DALYs averted), costs (both recurrent and capital), and service utilization patterns. Critical methodological assumptions included HEW time allocation to YFHS activities (15%, based on time–motion studies), a 1-year time horizon capturing 90% of DALY impacts, and disability weights sourced from GBD 2019 [20]. Sensitivity analyses tested these assumptions through probabilistic modeling and alternative scenarios.

#### 2.1.2. Epidemiological and Population Basis

The model used female adolescents aged 15–19 as the target population, reflecting the age group most at risk for adverse SRH outcomes. Disability weights were sourced from the Global Burden of Disease Study 2019 for key outcomes; adolescent pregnancy complications (e.g., eclampsia, hemorrhage), sexually transmitted infections (e.g., chlamydia, gonorrhea), unintended pregnancies and their psychosocial consequences, and epidemiological parameters, including incidence rates, service coverage, and health outcome probabilities, were drawn from the Ethiopian Demographic and Health Survey (EDHS), WHO, and peer-reviewed sources [28].

### 2.2. Study Area, Area, and Population

The study was conducted in Jimma Zone, Ethiopia, comparing intervention health posts (Shashamane, Ilala, Decha Nedi) with control health posts (Sombo Mana, Kaso Hixi, and Tikur Balto). Intervention sites had implemented a pilot program for youth-friendly health services (YFHS), while control sites provide routine care, enabling comparison of YFHS utilization and cost effectiveness. District selection was based on representativeness regarding demographics, service availability, and accessibility.

Ethiopia’s health delivery system operates through a structured three-tier model (primary, secondary, tertiary) to facilitate equitable health care access especially in rural areas, emphasizing primary healthcare service. At the primary level, healthcare is delivered via health posts and health centers.

Health Posts: Stuffed by Health Extension Workers (HEWs), these are community-based facilities providing preventive and basic curative services, with a focus on maternal and child health, immunization, family planning, and hygiene. Each health post serves about 3000–5000 people.

Health Centers: These facilities offer more comprehensive services, including outpatient care, basic laboratory services, and maternal health. Each health center serves about 25,000 people and supports 5–6 health posts.

#### Intervention Description

The intervention involved training HEWs to provide comprehensive youth-friendly sexual and reproductive health (SRH) services at rural health posts. This included contraceptive counseling and provision, antenatal care (ANC), screening for sexually transmitted infections (STIs), health education, and referral services. HEWs received specialized training in youth-friendly communication, confidentiality, and adolescent health management protocols. The control group consisted of health posts providing standard care without additional youth-friendly training.

### 2.3. Data Collection and Sources

Primary cost data were collected directly from participating health posts, including detailed records of staff time allocation, commodities, and supplies used. Secondary data were sourced from the published literature, government health databases, and demographic health surveys relevant to adolescent health in Ethiopia. Health outcome data, specifically DALYs averted, were estimated using epidemiological parameters from national and global health databases. Data cleaning and validation were cross-verified time-use data obtained from Health Extension Workers (HEWs) through structured interviews with supervisory staff; compared reported commodity usage against procurement and inventory logs from the participating health posts; and applied consistency checks to identify outliers or discrepancies across the six study sites (three intervention and three control sites).

#### 2.3.1. Costing Estimation

The micro-costing method, which focuses on detailed cost identification, measurement, quantification, and valuation, is employed to ensure precise economic evaluations [28,29,30]. This approach relies on comprehensive data collection from both primary sources, such as interviews and program records, and secondary sources, including payroll and procurement data, to enhance accuracy. Costs are systematically categorized into several areas: Personnel Costs, which encompass salaries and training; Supplies and Equipment Costs; Supervision and Program Management Costs; and Capital Costs, which cover buildings and equipment. Additionally, a time-adjusted cost analysis is conducted using a 3% discount rate to account for inflation and long-term investments. The micro-costing method is regarded as the gold standard in economic evaluations due to its ability to provide highly accurate cost data by meticulously analyzing individual cost components.

#### 2.3.2. Health Outcome Measurement

The primary outcome measure was the number of DALYs averted through the intervention compared to standard care. DALYs were calculated using disability weights and duration parameters obtained from the Global Burden of Disease Study and Ethiopian-specific health literature. Key health outcomes included reductions in adolescent pregnancies, improvements in antenatal care attendance, and increased contraceptive uptake.

#### 2.3.3. Sensitivity Analysis

To assess the robustness of the findings, sensitivity analyses were conducted. A one-way sensitivity analysis tested the effect of varying individual parameters, including costs, effectiveness rates, and service uptake. Additionally, probabilistic sensitivity analysis (PSA) was performed through Monte Carlo simulations (10,000 iterations) to account for joint uncertainty across multiple parameters. Results were reported using cost-effectiveness acceptability curves (CEACs).

### 2.4. Data Analysis

A comprehensive economic evaluation was cleaned, validated, and analyzed using a decision tree model in TreeAge software to assess the cost and cost-effectiveness of the YFHS intervention in health posts in Ethiopia. Descriptive statistics, including frequencies and percentages, summarized the characteristics of YFS utilization. Cross-tabulations compared findings between intervention and control districts, while bivariate analysis using Pearson’s chi-square tests examined relationships between variables. Multivariable regression analysis identified predictors of YFS utilization. Quantitative findings were triangulated with qualitative results to ensure a comprehensive understanding.

### 2.5. Ethical Considerations

Ethical approval was obtained from the Ethiopian Association of Anesthetists (EAA). Verbal consent was obtained from all participants, and confidentiality was maintained by using codes instead of personal identifiers.

## 3. Results

### 3.1. Costing Breakdown of YFHS Services

The costing analysis revealed a range of average unit costs across YFHS service categories, including family planning, maternal health, and disease control. Notably, services such as antenatal care (ANC) and contraceptive provision—although costlier—were associated with significantly greater uptake in the intervention group. For example, short-term injectable contraceptives had the highest per-client cost (USD 5.11) due to frequent dosing but also saw a 34% increase in utilization compared to control sites. In contrast methods such as condoms and long-term contraceptive implants were more cost efficient, with costs ranging from USD 1.93 to USD 3.01.

Maternal health interventions, such as antenatal care (ANC), postnatal care (PNC), and iron and folic acid supplementation, ranged between USD 1.63 and USD 1.86. Disease control interventions showed moderate costs, with HIV/AIDS and STI counseling between USD 2.38 and USD 2.44, while diabetes and hypertension risk assessments were lowest at USD 1.16. Nutrition services averaged USD 1.98 per client. Malaria prevention and HPV vaccination also had moderate unit costs (USD 1.39 and USD 1.73, respectively), while injury management was the lowest at just USD 0.22 (see Table 1).

### 3.2. Comparative Cost Breakdown Between Intervention and Control Groups

**Figure 2:**
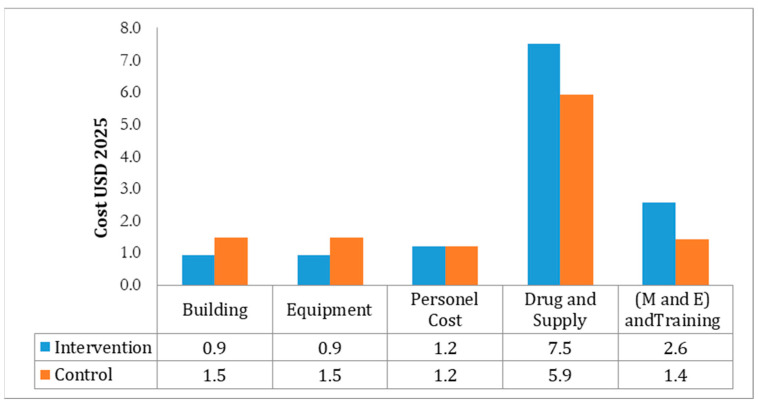
Distribution YFHS costs by ingredients approach for control and intervention groups, USD, Jimma Zone, 2025, Ethiopia.

In the control group, family planning services also emerged as the costliest, with injectables averaging USD 6.34 and condoms USD 2.41. Maternal health services had slightly higher unit costs in the control group (USD 2.01–2.25) than in the intervention group. Disease control services such as HIV/AIDS and STI education were particularly costly in the control group (USD 2.81 and USD 2.86). Overall, the intervention group provided broader service coverage (9854 clients vs. 2012.5 in the control group) but incurred higher total costs (USD 29,680 vs. USD 7519). Table 2 provides a detailed cost and coverage comparison. Figure 2 visually compares building, equipment, personnel, drug and supply, and M&E costs across both groups. These broader service coverages translated into substantially improved reproductive health outcomes, including greater ANC attendance and contraceptive uptake, which are examined in subsequent cost-effectiveness analyses (see Table 2).

### 3.3. Cost-Effectiveness of Youth-Friendly Health Services (YFHS) Program

The intervention group averted 52.11 DALYs, nearly double the 26.42 DALYs averted in the control group. This yielded an Incremental Cost-Effectiveness Ratio (ICER) of USD 25.50 per DALY averted. Notably, this falls well below Ethiopia’s GDP per capita in 2025 (estimated at USD 1027), in line with WHO guidance that deems such interventions ‘highly cost-effective’ when ICERs fall below the GDP per capita. This confirms the strong economic justification for scaling YFHS through HEWs at rural health posts (see Table 3).

### 3.4. Cost-Effectiveness and Impact Metrics

Table 4 summarizes key outcomes, showing that while the YFHS intervention incurred higher total costs (+USD 22,161), it delivered substantially greater health benefits—averting 25.69 additional DALYs (equivalent to 0.86 preventable deaths avoided) and achieving markedly higher service coverage (e.g., +49.5% for HPV vaccination). The intervention also reduced the cost per service user by $0.72. With an ICER of $25.50 per DALY averted, far below Ethiopia’s $3081 threshold, the model confirms high cost-efficiency (see Table 4).

### 3.5. Probabilistic Sensitivity Analysis (PSA)

**Figure 3:**
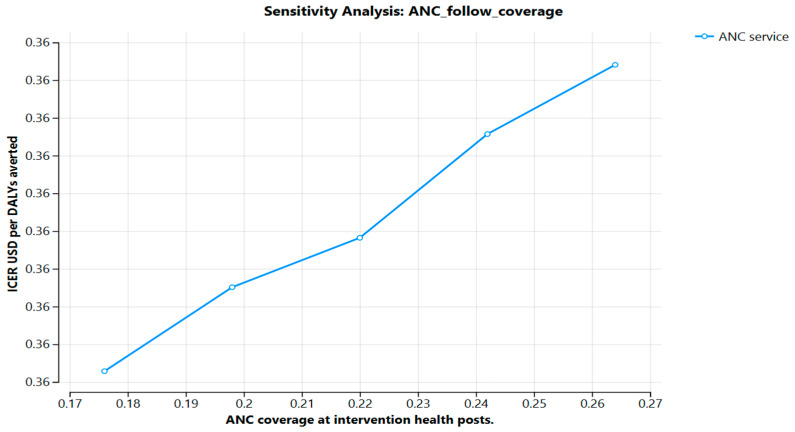
Probability of ANC coverage at intervention health posts, Jimma Zone, 2025, Ethiopia.

One-way sensitivity analyses showed that a USD 36 investment in ANC services led to a 27% increase in ANC uptake, and a USD 30 investment in family planning resulted in a 0.065% reduction in abortion-related mortality. These outcomes were the primary drivers of DALYs averted, highlighting the critical role of maternal care and contraceptive services in enhancing cost-effectiveness (see Figure 3).

**Figure 4:**
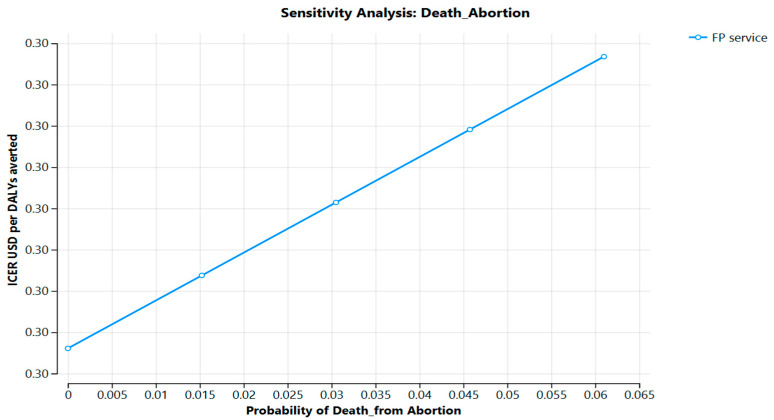
Probability of death from abortion, Jimma Zone, 2025, Ethiopia.

In the Figure 4 the probability of death from abortion in Jimma Zone is displayed.

### 3.6. Cost-Effectiveness Acceptability

**Figure 5:**
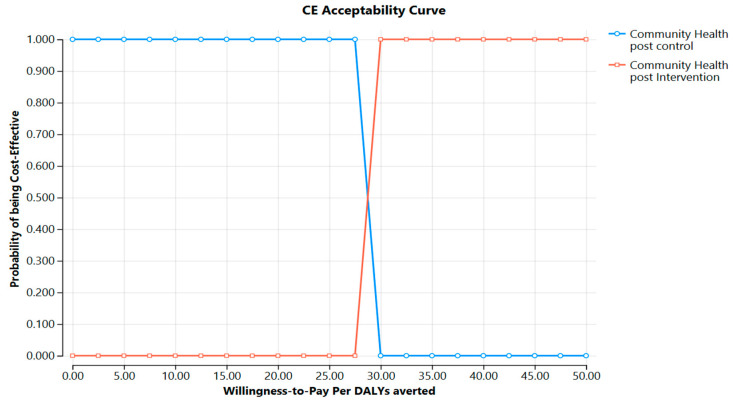
Cost-effectiveness acceptability curves YFHS program, Jimma Zone. 2025, Ethiopia.

Figure 5 displays the cost-effectiveness acceptability curve. At a willingness-to-pay (WTP) threshold of USD 25, the intervention and control arms have equal probabilities of being cost-effective. However, beyond USD 30, the intervention becomes the dominant strategy, given that the ICER remains far below Ethiopia’s GDP per capita, and the probability of the intervention being cost-effective remains consistently high (≥85%) at even modest willingness-to-pay thresholds, reaching 100% at USD 50, reinforcing the strong economic value of the intervention (see Figure 5).

#### Key Cost Drivers and Scenario Analysis

**Figure 6:**
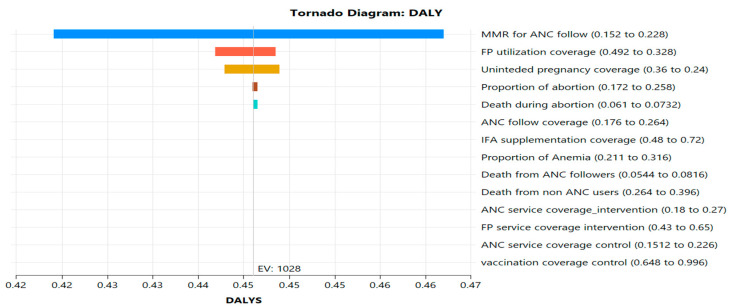
Tornado Diagram—ICER per DALYs averted, Jimma Zone. 2025, Ethiopia.

The tornado diagram (Figure 6) highlights the most influential variables on the ICER: maternal mortality reduction through ANC follow-up had the greatest impact, followed by family planning uptake, unintended pregnancy prevention, and abortion-related mortality. Moderate contributors included IFA supplementation and anemia prevalence, while vaccination and ANC service coverage in control sites had minimal effect. These findings further support prioritizing ANC and family planning as strategic investment points for improving adolescent health outcomes and reducing DALYs at a low incremental cost.

## 4. Discussion

This study aimed to evaluate the cost-effectiveness of youth-friendly health services (YFHS) delivered by Health Extension Workers (HEWs) at health posts compared to standard service delivery in rural Ethiopia. The findings clearly demonstrate that the YFHS intervention not only improved service coverage but also yielded substantially better health outcomes, most notably increased contraceptive use, higher antenatal care (ANC) uptake, and a measurable reduction in abortion-related mortality. A central finding of this evaluation is the incremental cost-effectiveness ratio (ICER) of USD 25.50 per DALY averted. According to World Health Organization (WHO) guidelines, health interventions are considered highly cost-effective if the ICER is less than a country’s GDP per capita. Ethiopia’s GDP per capita in 2025 is estimated at USD 1027, placing the YFHS intervention well below this threshold. This positioning confirms the intervention as a high-value investment for Ethiopia’s health system, particularly for addressing adolescent sexual and reproductive health (SRH) inequities in resource-limited rural settings.

This analysis provides a pioneering economic evaluation specific to Ethiopia, addressing a critical evidence gap in adolescent health service provision at the primary care level. By integrating detailed primary cost data and robust economic modeling, this study offers valuable insights into the scalability of YFHS through Ethiopia’s Health Extension Program (HEP). The significance lies in its potential to influence policy makers to allocate resources effectively towards youth-targeted health initiatives.

These economic findings align with prior analyses of Ethiopia’s primary healthcare system. In Ethiopia, a cost-effectiveness analysis of the Health Extension Program, which includes community-based interventions such as the work of HEWs, has also shown that maternal health and family planning services are generally cost-effective [21]. This supports the findings of the current study, where interventions like antenatal care and contraceptive provision were relatively low-cost and impactful. While these studies align with the current findings, variations exist in cost structures due to differences in economic settings, healthcare infrastructure, and funding sources.

These results are consistent with global and regional evidence that youth-focused health strategies, especially those involving trained community health workers, are both impactful and cost-effective. For instance, a cost-effectiveness study in Moldova by Kempers et al. (2014) found that youth health centers providing SRH services significantly reduced STIs and unintended pregnancies at relatively low cost [12,17]. Similar to this study, it demonstrated that interventions focusing on health worker training enhanced service utilization. Similarly, research in sub-Saharan Africa highlights the importance of healthcare provider training and adolescent-specific service delivery in increasing health service uptake and outcomes [14,22]. Their analysis, which showed a significant reliance on external funding and highlighted personnel salaries as a major cost driver, aligns closely with our findings regarding the economic dynamics of implementing youth-friendly services.

Similarly, research in Botswana highlighted the substantial long-term economic benefits of youth interventions through improved educational outcomes, which, in turn, reduced HIV infections and other adverse health outcomes [23]. While our study focused explicitly on health outcomes without direct consideration of educational effects, both studies underscore the critical long-term returns of investing in adolescent-focused interventions. While the long-term educational and economic benefits were not directly assessed in our model, the short-term DALY reductions observed suggest high potential for long-term value, reinforcing the importance of sustained investment.

The health benefits demonstrated by the YFHS intervention extend beyond clinical outcomes and represent a substantial investment in adolescent human capital. By averting 52.11 DALYs—primarily through reductions in unintended pregnancies, improved antenatal care attendance, and lower abortion-related mortality—this program helps adolescents, especially girls, remain in school and avoid early parenthood, which are critical determinants of their long-term socioeconomic trajectories. Early childbearing is strongly associated with school dropout, diminished lifetime earnings, and intergenerational poverty, particularly in rural Ethiopia where access to educational and economic opportunities is already limited. Research in sub-Saharan Africa shows that each additional year of secondary schooling reduces the risk of early pregnancy and improves lifetime income potential, particularly for girls [12,23]. Therefore, cost-effective YFHS interventions like the one evaluated in this study do not merely improve immediate health outcomes—they also enhance future workforce productivity and national development prospects by safeguarding adolescents’ ability to accumulate skills, complete education, and participate in the labor market. Framing YFHS through the lens of human capital theory underscores its strategic value not only for health equity but also for inclusive economic growth and social development in Ethiopia.

However, there are notable differences. For instance, the Nicaraguan voucher scheme analyzed by Borghi et al. (2005) showed significant cost-effectiveness in STI management, though primarily through private-sector engagement [24,25,30]. While our findings affirm the cost-effectiveness of integrating youth-friendly health services (YFHS) into the existing public-sector Health Extension Worker (HEW) model in Ethiopia, it is instructive to contrast this approach with alternative delivery mechanisms in other resource-limited settings. For example, the private-sector voucher scheme for adolescent SRH services in Nicaragua reported a lower cost per DALY averted (USD 19.7) but relied on subsidized access through private providers and external donor funding [13,14,24]. In contrast, the HEW model is embedded within Ethiopia’s decentralized primary healthcare system and leverages existing public health infrastructure, making it potentially more scalable and sustainable. However, private-sector models may offer greater flexibility, faster innovation cycles, and potentially higher service responsiveness, especially in urban contexts.

A significant strength of this study includes its use of primary data collection, detailed costing methodology, and extensive sensitivity analyses, which enhance the robustness and applicability of findings. However, limitations include the geographical constraint, limiting generalizability beyond Jimma Zone, and reliance on secondary sources for certain costs and health parameters. Furthermore, while the short-term nature of the data provides immediate insights, it may not fully capture long-term economic implications. Future research should broaden the geographic scope, include long-term follow-up for cost-effectiveness, and explore qualitative dimensions influencing adolescent health service utilization. These features strengthen the policy relevance of our findings, particularly as Ethiopia considers expanding adolescent-focused services under its Essential Health Services Package (EHSP).

Given the demonstrated cost-effectiveness, it is plausible to speculate that scaling similar interventions across Ethiopia could significantly reduce national health burdens, particularly those associated with adolescent pregnancies and sexually transmitted diseases. Overall, these findings underscore the strong value-for-money and health return of YFHS delivery through HEWs at the primary care level. The intervention supports national policy goals under the Ethiopian Adolescent and Youth Health Strategy and has the potential to significantly reduce adolescent SRH-related health burdens across Ethiopia. Health policymakers should thus consider embedding YFHS into Ethiopia’s Essential Health Services Package (EHSP) and prioritize investments in youth-specific training, supportive supervision, and commodity supply chains to scale YFHS programs nationally.

## 5. Conclusions

In conclusion, providing youth-friendly health services (YFHS) through trained HEWs at rural health posts is a highly cost-effective strategy for improving adolescent sexual and reproductive health (SRH) outcomes in Ethiopia. The intervention led to measurable improvements, including a 27% increase in antenatal care uptake, a 34% rise in contraceptive use, and reductions in abortion-related mortality while achieving an incremental cost-effectiveness ratio (ICER) of USD 25.50 per DALY averted. This is substantially below Ethiopia’s GDP per capita threshold of USD 1027, confirming the intervention’s high economic value according to WHO standards.

By leveraging the existing Health Extension Program infrastructure, the YFHS model demonstrates scalability and sustainability, requiring only modest investment in training, resource mobilizations and supervision. Given the strong returns in both health outcomes and cost efficiency, YFHS should be prioritized for national scale-up and integration into Ethiopia’s Essential Health Services Package (EHSP); integrate adolescent health competencies into HEW training; embed adolescent-specific indicators (e.g., age-disaggregated data, privacy) into DHIS2 for real-time monitoring; secure sustainable financing via the 2025 EHSP with dedicated budgets; and establish accountability through MoH oversight, youth advisory committees, and woreda-level incentives. Strategic investments in adolescent-focused service delivery have the potential to substantially reduce preventable morbidity and mortality while advancing health equity and universal health coverage goals.

## Figures and Tables

**Table 1:** Costing breakdown of youth-friendly health services (YFHS) program interventions in woreda, (intervention arm), Jimma Zone, 2025, Ethiopia.

Program	Service Type	Building	Equipment	Personnel	Supply	M and E and Training
Family Planning	Provision of short-term contraceptive (COC)	1.92	0.12	0.06	0.76	0.16
Family Planning	Provision of short-term contraceptive (condom)	0.96	0.00	0.06	0.76	0.16
Family Planning	Provision of short-term contraceptive (injectables)	3.83	0.25	0.11	0.76	0.16
Family Planning	Provision of long-term contraceptive (implant)	0.96	0.02	0.03	0.76	0.16
Maternal Health	Provision of ANC (three)	0.96	0.07	0.11	0.55	0.16
Maternal Health	Provision of ANC (seven)	0.96	0.07	0.03	0.55	0.16
Maternal Health	Provision of TD 2+ vaccination for pregnant	0.96	0.10	0.03	0.48	0.16
Maternal Health	Provision of TD2+ vaccination for non-pregnant	0.96	0.10	0.07	0.48	0.16
Maternal Health	Provision of iron and folic acid supplementation for the mother	0.96	0.04	0.07	0.40	0.16
Maternal Health	Provision of early postnatal care (PNC)	0.96	0.06	0.07	0.55	0.16
Disease Control	Prevention of DM and hypertension	0.96	0.00	0.07	0.03	0.16
Disease Control	HIV/AIDs health education counseling	1.92	0.08	0.09	0.14	0.16
Disease Control	STI HE/counseling	1.92	0.08	0.14	0.14	0.16
Nutrition	Nutritional counseling	0.96	0.04	0.06	0.76	0.16
	Injury management	0.00	0.05	0.00	0.00	0.16
Malaria Prevention and Control	Counseling and psychosocial assessment	0.96	0.08	0.06	0.14	0.16
Disease Control	Referral for HIV and other STI screening	0.96	0.01	0.11	0.14	0.16
Disease Control	HPV vaccination	0.96	0.02	0.11	0.48	0.16

**Table 2:** Costing breakdown of youth-friendly health services (YFHS) program interventions in (control arm), Jimma Zone, 2025, Ethiopia.

Program	Service Type	Building	Equipment	Personnel	Supply	M and E and Training
Family Planning	Provision of short-term contraceptive (COC)	0.90	0.13	0.06	1.08	0.07
Family Planning	Provision of short-term contraceptive (condom)	00	0.00	0.06	1.08	0.07
Family Planning	Provision of short-term contraceptive (injectables)	4.79	0.28	0.11	1.08	0.07
Family Planning	Provision of long-term contraceptive (implant)	1.20	0.02	0.03	1.08	0.07
Maternal Health	Provision of ANC (three)	1.20	0.08	0.11	0.78	0.07
Maternal Health	Provision of ANC (seven)	1.20	0.08	0.03	0.78	0.07
Maternal Health	Provision of TD2+ vaccination for pregnant	1.20	0.11	0.03	0.66	0.07
Maternal Health	Provision of TD2+ vaccination for non-pregnant	1.20	0.11	0.07	0.66	0.07
Maternal Health	Provision of iron and folic acid supplementation for the mother	1.20	0.05	0.07	0.63	0.07
Maternal Health	Provision of Early Postnatal care (PNC)	1.20	0.07	0.07	0.78	0.07
Disease Control	Risk assessment and prevention of DM and hypertension	0	0.00	0.00	00	0.07
Disease Control	HIV/AIDs health education counseling	2.39	0.09	0.09	0.16	0.07
Disease Control	STI health education/counseling	2.39	0.09	0.14	0.16	0.07
Nutrition	Nutritional counseling	1.20	0.05	0.06	1.08	0.07
	Injury management	0.00	0.00	0.00		0.07
Malaria Prevention and Control	Counselling and psychosocial assessment	1.20	0.09	0.06	0.16	0.07
Disease Control	Referral for HIV and other STI screening	1.20	0.09	0.11	0.16	0.07
Disease Control	HPV vaccination	1.20	0.11	0.11	0.66	0.07

**Table 3:** Cost-effectiveness ratio of YFHS per DALYs averted, Jimma Zone, 2025, Ethiopia.

Cost, Effectiveness, Average and Incremental Cost-Effectiveness Ratio per DALYs Averted.					
Strategy	Cost (USD)	Incremental Cost	Eff (DALYs)	Incremental Eff	ICER
Health post control	4.96		26.42		
Health post intervention	6.25	1.26	52.11	25.69	25.50

**Table 4:** Comparative outcomes of YFHS intervention vs. standard care.

Indicator	Intervention Group	Control Group	Net Difference
Costs (USD)			
Total program cost	29,680	7519	+22,161
Cost per service user	3.01	3.73	−0.72
Health Outcomes			
DALYs averted (total)	52.11	26.42	+25.69
Years of healthy life gained ^1^	52.11	26.42	+25.69
Equivalent deaths avoided ^2^	1.74	0.88	+0.86
Service Coverage			
ANC service utilization	22.4%	18.9%	+3.5%
Family planning utilization	54.9%	50.7%	+4.2%
HPV vaccination coverage	74.0%	24.5%	+49.5%
Cost-effectiveness			
ICER (USD per DALY averted)	25.50	-	-

Notes: ^1^ Years of healthy life gained = DALYs averted (1 DALY = 1 year of healthy life). ^2^ Deaths avoided = DALYs averted/30 (WHO, 2014 standard conversion: 1 death averted ≈ 30 DALYs).

## Data Availability

The data presented in this study are available based on request from the corresponding author. The data is not publicly available due to privacy reasons.
